# Social Determinants of Active Aging: Differences in Mortality and the Loss of Healthy Life between Different Income Levels among Older Japanese in the AGES Cohort Study

**DOI:** 10.1155/2012/701583

**Published:** 2012-09-18

**Authors:** Hiroshi Hirai, Katsunori Kondo, Ichiro Kawachi

**Affiliations:** ^1^Department of Civil Environmental Engineering, Faculty of Engineering, Iwate University, Iwate, Morioka 020-8551, Japan; ^2^Center for Well-Being and Society, Nihon Fukushi University, Nagoya 460-0012, Japan; ^3^Department of Society, Human Development, and Health, Harvard School of Public Health, Boston, MA 02115, USA

## Abstract

We examined the relationship between income, mortality, and loss of years of healthy life in a sample of older persons in Japan. We analyzed 22,829 persons aged 65 or older who were functionally independent at baseline as a part of the Aichi Gerontological Evaluation Study (AGES). Two outcome measures were adopted, mortality and loss of healthy life. Independent variables were income level and age. The occurrence of mortality and need for care during these 1,461 days were tracked. Cox regressions were used to calculate the hazard ratio for mortality and loss of healthy life by income level. We found that people with lower incomes were more likely than those with higher incomes to report worse health. For the overall sample, using the governmental administrative data, the hazard ratios of mortality and loss of healthy life-years comparing the lowest to the highest income level were 3.50 for men and 2.48 for women for mortality and 3.71 for men and 2.27 for women for loss of healthy life. When only those who responded to questions about income on the mail survey were included in the analysis, the relationships became weaker and lost statistical significance.

## 1. Introduction

There is a well-established inverse relationship between income and health [1–7]. However, many of the studies reporting on this relationship have used mortality as an indicator of health. In evaluating health, the World Health Organization recommends using indicators that reflect quality of life (QOL), such as healthy life expectancy, which measures active aging. Active aging aims to extend healthy life expectancy and quality of life in older persons, and the quality of life is largely determined by its ability to maintain autonomy and independence [8]. Fewer studies use active aging as an endpoint because these measures, unlike measures of mortality, require investigation into the physical and cognitive functioning of surviving participants. As a result, in large-scale cohort studies it is much more difficult and costly to follow functional status over a long period than to simply follow mortality.

Measuring income is a difficult issue in studies that investigate the relationship between income and health. Individuals with lower socioeconomic status (SES) tend to be less likely to respond to surveys by mail or similar means. In addition, income data are often unreliable or missing for a large part of the sample [9]. Therefore, lower income groups, which are predicted to be the least healthy, are not well represented. This underrepresentation gives rise to the possibility of underestimating the inequality in health. However, while this problem has been acknowledged, there are no studies that have compared analyses using government statistical data, which have almost no missing data, and analyses of survey respondent data to investigate the existence and size of underevaluation of the income inequality. 

Fortunately, we have been able to overcome the issues involved in conducting a large-scale cohort study that monitors functional status and obtaining income data from both surveys and local government statistics. The Japanese government introduced a public long-term care insurance system in 2000. Insurance applicants are assessed using standard criteria for physical and cognitive functioning to determine eligibility. Long-term care insurance premiums are imposed on everyone 65 years of age or older who is insured under the long-term care insurance system. These premiums are set according to income level, which is determined by the municipality in which the insured person lives. 

In this study, we used representative sample data of five municipalities that contains information on physical and cognitive functional declines and income data from the government to examine the relationship between health and income. In addition, we conducted a survey by mail, and using this government data and data on responses to income items in the mail survey, we investigated the effect of differential response in income self-reports among the elderly. 

Measuring the inequality in health using Japanese data is also thought to be meaningful in other ways, as the healthy life expectancy in Japan is among the longest in the world [10]. In addition to background factors, such as improved medical services and dietary habits that come with universal healthcare and economic growth, the equitableness of Japanese society, with small inequalities among people, has attracted attention as a possible reason [11, 12]. In a recent meta-analysis, health levels are reported to be lower among people living in countries with a large Gini coefficient and very unequal income distributions [13]. 

However, since the 1990s, the Gini coefficient has been rising in Japan as the income inequality has widened. There is concern that in the coming years the inequality in health will continue to grow in Japan, making it similar to other developed countries. However, there are particularly few investigations on the relationship between income and health in Japan. Moreover, most of the studies that have been conducted on this subject are ecological studies, such as the studies by Fukuda and colleagues [14, 15] that show a relationship between income level and mortality in communities. The only cohort study that we are aware of was conducted by Kimura and colleagues [16]. This study demonstrated an association with mortality using scores (0–6) prepared from six dichotomous indicators related to income. No studies have investigated the relationship between quantitative income and health at the individual level [17]. 

## 2. Objectives

The purpose of this study was to investigate the relationship between health and income using a prospective cohort study design. The investigation was dually focused and included (1) an investigation of the relationship between mortality and income and loss of healthy life and income; (2) a comparison of responders of a self-administered mail survey to a sample of elderly people, including those who did not respond to the survey, to investigate differences in the income-health relationship in the two samples. 

## 3. Materials and Methods

### 3.1. Study Population

The present study is based on data from the Aichi Gerontological Evaluation Study Project. The project is an ongoing prospective cohort study that started in two municipalities in Aichi Prefecture, Japan, in 1999. The project focuses on elderly people aged 65 years and older who are independent in physical and cognitive functioning. In second wave surveys of this project, research results are being accumulated with a focus on cross-sectional data obtained in fiscal 2003 from 15 municipalities in three prefectures [18–21]. Currently, follow-up data on certification of long-term care need and mortality are being obtained for the municipalities that cooperated in this study and from among the municipalities that are the subjects of second wave surveys. There are several published articles using this cohort data [22–24]. 

The present study looked at elderly people 65 years of age or older living in five municipalities that provided income data. Ethical approval for the study was obtained from the Nihon Fukushi University ethics committee. 

### 3.2. Participants

Self-administered questionnaires completed by elderly people 65 and older living in one of five participating municipalities were collected by mail in October 2003. The questionnaire included items that asked about the physical, mental, and social situation of respondents. Of the 24,374 people selected to receive the survey by the municipalities, those who had been certified as needing long-term care as of October 31, 2003 were excluded, and the remaining 22,829 people were included as subjects in the present analysis. The mean age ± SD of the subjects was 73 ± 6.3 years, and there were 10,290 men and 12,593 women. 

The age and income level distribution of the entire sample is shown in Table 2. The standards were the same for men and women, but the distribution for men and women differed. More than 20% of men were in the fifth level and more than 50% were in the fourth and fifth levels combined. In comparison, less than 10% of the women were in the fourth and fifth levels combined. Here are some reasons for the gender difference in income. First, the greater part of Japanese women was full-time housewives. Full-time housewives were not obliged to enroll in the national pension scheme until 1985, so some part of them had not participated in the national pension and they receive lower pension benefits. Second, the percentage of older women living alone exceeds that of men. According to the 2000 national census, the living alone rate for older women is 17.9 percent, compared to 8.0 percent for men. Single households have considerably lower household income than other household type.  Table 3 shows the number of people and percentage that responded to the survey and to questions about income by age and income level. There was a tendency for both men and women in high income groups to have a high response rate. 

### 3.3. Measures

#### 3.3.1. Dependent Variables

Two outcome measures were adopted, mortality and loss of healthy life. Data were collected from the public long-term care insurance database maintained by each participating municipality. Mortality was ascertained using the insured person list of the public long-term care insurance. Loss of healthy life was defined as mortality, functional decline, or cognitive impairment. The condition of “functional decline or cognitive impairment” means a condition assumed to require care on a continual and steady basis for the whole or a part of basic movements in daily activities. Functional decline or cognitive impairment assessed using standards presented by the national government, an examination of mental and physical status based on a visiting survey to maintain objectivity and reliability and make a screening judgment based on the opinions of a regular doctor [25].

#### 3.3.2. Independent Variables

Income level was based on calculations used to determine long-term care insurance premiums, since long-term care insurance premiums in Japan are determined based on income level (Table 1). Subjects who are exempt from the municipal residence tax earn an income of less than 1.25 million yen according to criteria set in 2003. 

#### 3.3.3. Statistical Methods

The study sample were followed from November 1, 2003 to October 31, 2007. The occurrence of mortality and need for care during these 1,461 days were tracked. Cox proportional hazard models were used to calculate the hazard ratio (HR) and 95% confidence intervals (CIs) for mortality and loss of healthy life by income level. All analyses were stratified by gender. In addition, an analysis including only subjects who responded to questions about income on the self-administered survey was conducted, and results were compared. 

## 4. Results

### 4.1. Aggregate Totals, Number of Outcome Events, and Rates of Mortality and Certification of Long-Term Care Need during the Follow-Up Period

There were 1,328 deaths among men and 944 deaths among women in over 38,442 person-years of observation in men and 48,120 person-years in women. Loss of healthy life was observed in 2,157 men and 2,636 women in over 36,565 person-years of observation in men and 44,483 person-years in women. Followup was not possible for 113 men and 162 women either because they moved out of the area or for other reasons. 

### 4.2. Main Results

Table 4 shows the age-adjusted hazard ratios for community-dwelling independent elderly. In men, using death as the endpoint and income level 5 as the reference, hazard ratios reached statistical significance from level 3 (HR 1.55, 95% CI 1.31–1.84) to level 1 (HR 3.50, 95% CI 1.91–6.42). When loss of healthy life was the endpoint, hazard ratios were statistically significant for all income levels compared to income level 5; from level 4 (HR 1.23, 95% CI 1.07–1.41) to level 1 (HR 3.71, 95% CI 2.24–6.13). Among women, when mortality was the endpoint, hazard ratios were statistically significant for level 1 compared to level 5 (HR 2.48, 95% CI 1.09–5.67). When loss of healthy life was the endpoint, hazard ratios were statistically significant for level 2 (HR 1.41, 95% CI 1.10–1.81) and level 1 (HR 2.27, 95% CI 1.43–3.63) compared to level 5.

In contrast, when only those who responded to questions about income on the mail survey were included in the analysis, among men, the hazard ratio, comparing the lowest to the highest income level, was smaller than with the full sample, and, among women, the relationship between income and health was no longer significant.

## 5. Discussion

### 5.1. Key Results

Our study analyzed government health data of independent elderly, and we found that people with lower incomes were more likely than people with higher incomes to lose their health when using both mortality and loss of healthy life as endpoints. The hazard ratio comparing the lowest income level to the highest income level was 3.50 for men and 2.48 for women when using mortality as an endpoint and 3.71 for men and 2.27 for women when using loss of healthy life as an endpoint. When only those who responded to questions about income on the mail survey were included in the analysis, the relationship became weaker. 

### 5.2. Limitations

This study has the following limitations. The government data only included information on the taxable income of the individual. Thus, the income of other household members remained unclear. Further, the study only considered income, and accumulated wealth or assets were not included in the analysis. Therefore, in some cases, these data may not reflect actual economic affluence. In addition, since data other than income, age, and sex were not obtained, there may have been confounding factors that we were unable to adjust for. Hence, these results do not mean a causal link but an observational relationship including confounders such as education level and behavioural risk factors.

### 5.3. Interpretation

This study provides several findings not reported in previous studies. First, the results of this study verifies, using a large dataset with a high follow-up rate (98.8%), the relationship between low income, mortality, and risk of decreased physical and cognitive functioning in a sample of older adults. It is difficult to ensure a high follow-up rate in cohort studies that use functional decline as the endpoint. For example, Beydoun and Popkin [4] followed a sample of 976 out of the original 1,385 subjects (follow-up rate: 70.5%) for three years, Lynch and colleagues [26] followed a sample of 1,124 out of 1,799 subjects (follow-up rate: 62.4%) for 11 years, and Guralnik and Kaplan [5] followed a sample of 496 out of 2,392 subjects (follow-up rate: 20.7%) for 19 years. Second, by comparing all independent elderly included in the government data to those who responded to questions on income in a mail survey, we demonstrated that measurement of the inequality in health is underestimated because individuals with low SES are less likely respond to self-administered surveys. 

The finding that the relationship between SES and health indicators is stronger for men than women has been reported in many studies. There are also reports, such as that of Bassuk and colleagues [27], of a strong association between income and mortality in women. In the present study, significantly larger hazard ratios were obtained for men compared to those for women for both mortality and loss of healthy life. However, there are large differences between men and women in the distribution of income, and so when comparing the size of hazard ratios in men and women, due consideration should be given in the comparison of percentile in the groups from which the reference and HR are obtained. Men in the fifth income level corresponding to high income were the top 23.5 percentiles, while women were the top 3.3 percentiles. Since women are understood to be in a higher level, hazard ratios would tend to be larger in women than in men when obtaining the hazard ratios of the lower levels. However, in fact, the hazard ratios for women are smaller than in men. If the top 20 percentiles are taken as the reference for women the same as in men, hazard ratios may become even smaller. Thus, the relation between income and health is thought to be stronger in men than in women.

Our results demonstrated that men and women with low income levels are less healthy than people with high income levels. We compared our results to those of previous studies conducted in other countries. Since income distributions differed among studies, we took this into consideration when comparing results. In the present study, the percentage of people with the lowest income (level 1) was small. Further, people in income level 1 may be more likely to receive certification of long-term care need compared to people of income level 2 or higher, since people receiving public assistance do not pay for care services. As a result, we used income level 2 for comparison, which corresponds roughly to the 20th percentile from the bottom in men and the 30th percentile in women.

Looking at mortality in this study, the HR was 1.53 in men of the level 2 group, which corresponded to 78.7–99.6% from the top, compared to the reference category (level 5) which is 0–23.5% from the top. In previous studies listed in Table 5 (see Osler et al. [28] and Manor et al. [29]) the top 25% was used as the reference group, similar to the present study. Osler and colleagues reported a slightly higher HR of 1.92 for a comparison of similar groups. Manor and colleagues reported an HR of 1.61 for the 75–100 percentiles, similar to our level 2 group, which can be compared to the HR of 1.53 found in our study. In women, the HR comparing the level 2 group, which corresponded to 70.1–99.6 percentiles, to the reference category, which corresponded to the 3.3 percentile, was 1.54. In a study that looked at women 65 years of age, Bassuk and colleagues [27] reported an HR of 2.13 for the 49–100 percentiles compared to the top 5.9%, similar to the reference group used in the present study. Finally, Martikainen and colleagues [9] reported an HR of 1.47 for 70–80 percentiles compared to the top 10% and an HR of 1.51 for the 80–90 percentiles compared to the top 10%. These findings are similar to those found in the present study, as we found an HR of 1.54 for the level 2 group, which corresponds to the 70.1–99.6 percentiles. We take these similarities between our results and those of previous studies as evidence that, while the inequality in health in Japan is not large compared to other countries, neither is it particularly small. 

In the present study, the HR for loss of healthy life among men that compared the 78.7–99.6 percentiles to the top 23.5 percent was 1.73. For women, the HR comparing the bottom 70.1–99.6 percentiles to the top 2.3 percent was 1.41. In contrast, Beydoun and Popkin [4], who looked at declining ADL or IADL, reported an HR of 1.69 when comparing the 70–100 percentiles to the top 20 percent. 

When comparing our results for all independent elderly to our results for those who responded to questions about income in the mail survey, we found that the hazard ratios were lower for those that responded to the survey for both mortality and loss of healthy life, and, for women that responded to the survey, hazard ratios did not reach statistical significance. These results provide evidence that, when evaluating the inequality in health, underestimation may occur if subjects are limited to people who respond to income items on self-administered mail surveys, and the real inequality in health may not be detected. 

Hirdes and Forbes [2] conducted a study using data from the 1995 National Livelihood Survey in Japan and found a lack of support for the relative income hypothesis, which is consistent with the view expressed by Marmot and Smith [11] and Wilkinson [12]. However, Oshio and Kobayashi [30], using National Livelihood Survey data from 2004, and Ichida and colleagues [19], using data from a 2003 independent survey, found that the relative income hypothesis was supported. These differences may be because Japan has entered a period of breakdown in its traditional social structure (Kagamimori et al. [17]), and socioeconomic inequality in Japan seems to be associated with more serious health consequences. The present study used 2003 data. The inequality in health in Japan may be growing larger as a result of the recent expansions in the income inequality. 

## 6. Conclusion

We revealed that the lower income was a significant negative social determinant of active aging with a hazard ratio of 3.71–2.27 for the lowest income people. This was the first cohort study in Japan to examine the relationship between income, mortality, and declines in physical and cognitive functioning using individual level local government data with a high follow-up rate. In addition, we demonstrated that, due to missing data, the inequality in health may be underestimated when data are gathered using self-administered mail surveys.

It is meaningful that the present results, which avoided underestimation of the inequality in health, provided evidence that the inequality in health among older Japanese is similar to that found in other countries. While there is great variety in the measures taken by governments against the inequality in health, from ignoring the inequality altogether to adopting comprehensive, coordinated policies, in Japan and many other countries, measurement of the inequality in health is inadequate (Whitehead [31], Kondo [32]). Thus, measuring the inequality in health is an important first step in beginning to resolve it. Measuring the inequality in health in future surveys in all parts of the country and clarifying the status of these inequalities in health will provide clues for taking the next step necessary to correct the inequality in health and facilitate active aging. 

## Figures and Tables

**Table 1:** Income level.

Income level	Eligible persons	Premium
Level 1	Public assistance recipients	Basic amount × 0.5
	Municipal tax-exempted households and old-age welfare	
	Pension recipients	
Level 2	Municipal tax-exempted households	Basic amount × 0.75
Level 3	Municipal tax-exempted persons	Basic amount × 1
Level 4	Municipal tax payer (the insured person's total amount of income is less than 2,500,000 yen)	Basic amount × 1.25
Level 5	Municipal tax payer (the insured person's total amount of income is 2,500,000 yen or more)	Basic amount × 1.5

**Table 2:** Baseline distribution of data on age and income level.

		Men			Women	
	NO.	%	Cumulative %	NO.	%	Cumulative %
Total	10290	100.0		12539	100.0	

Age						
65–69	3716	36.1	36.1	3908	31.2	31.2
70–74	3184	30.9	67.1	3452	27.5	58.7
75–79	2060	20.0	87.1	2709	21.6	80.3
80–84	895	8.7	95.8	1490	11.9	92.2
85+	435	4.2	100.0	980	7.8	100.0
Income level						
Level 5 (high)	2417	23.5	23.5	413	3.3	3.3
Level 4	3113	30.3	53.7	480	3.8	7.1
Level 3	2568	25.0	78.7	7900	63.0	70.1
Level 2	2150	20.9	99.6	3691	29.4	99.6
Level 1 (low)	42	0.4	100.0	55	0.4	100.0

**Table 3:** Respondents rate for mail survey and item about income.

		Whole sample (A)	Number of respondents for mail survey (B)	Respondents rate (B/A,%)	Number of respondents for item about income (C)	Respondents rate for item about income (C/A, %)
	Total	10290	5513	53.6	4824	46.9
	Age					
	65–69	3716	2016	54.3	1854	49.9
	70–74	3184	1666	52.3	1469	46.1
	75–79	2060	1112	54.0	939	45.6
	80–84	895	498	55.6	389	43.5
Men	85+	435	221	50.8	173	39.8
	Income level					
	Level 5 (high)	2417	1434	59.3	1332	55.1
	Level 4	3113	1920	61.7	1744	56.0
	Level 3	2568	1146	44.6	890	34.7
	Level 2	2150	1007	46.8	854	39.7
	Level 1 (low)	42	6	14.3	4	9.5

	Total	12539	6375	50.8	4467	35.6
	Age					
	65–69	3908	2012	51.5	1599	40.9
	70–74	3452	1767	51.2	1286	37.3
	75–79	2709	1446	53.4	934	34.5
	80–84	1490	754	50.6	417	28.0
Women	85+	980	396	40.4	231	23.6
	Income level					
	Level 5 ( high)	413	223	54.0	172	41.6
	Level 4	480	266	55.4	209	43.5
	Level 3	7900	4042	51.2	2772	35.1
	Level 2	3691	1827	49.5	1308	35.4
	Level 1 (low)	55	17	30.9	6	10.9

**Table 4:** Age-adjusted hazard ratio for mortality and loss of healthy life.

				Mortality				Loss of healthy life			
		*n*	%	Hazard ratio	95% confidence interval		*P*	Hazard ratio	95% confidence interval		*P*
					Lower	Upper			Lower	Upper	
Sample subjects are all community-dwelling independent elderly											

	Income level										
	Level 5	2417	23.5	1.00				1.00			
	Level 4	3113	30.3	1.17	0.99	1.40	0.073	1.23	1.07	1.41	0.004
Men	Level 3	2568	25.0	1.55	1.31	1.84	<0.001	1.54	1.35	1.77	<0.001
	Level 2	2150	20.9	1.53	1.28	1.82	<0.001	1.73	1.51	1.99	<0.001
	Level 1	42	0.4	3.50	1.91	6.42	<0.001	3.71	2.24	6.13	<0.001

	Income level										
	Level 5	413	3.3	1.00				1.00			
	Level 4	480	3.8	1.44	0.79	2.62	0.231	1.17	0.84	1.64	0.352
Women	Level 3	7900	63.0	1.42	0.90	2.25	0.129	1.12	0.88	1.43	0.363
	Level 2	3691	29.4	1.54	0.97	2.45	0.069	1.41	1.10	1.81	0.007
	Level 1	55	0.4	2.48	1.09	5.67	0.031	2.27	1.43	3.63	0.001

Sample subjects are limited to those who responded about income in the mail survey											

	Income level										
	Level 5	1332	27.6	1.00				1.00			
	Level 4	1744	36.2	1.03	0.81	1.32	0.790	1.12	0.92	1.37	0.242
Men	Level 3	890	18.4	1.42	1.10	1.84	0.008	1.47	1.20	1.82	<0.001
	Level 2	854	17.7	1.24	0.94	1.63	0.125	1.59	1.29	1.97	<0.001
	Level 1	4	0.1	—	—	—	—	2.16	0.30	15.42	0.444

	Income level										
	Level 5	172	3.9	1.00				1.00			
	Level 4	209	4.7	1.86	0.62	5.56	0.267	1.12	0.65	1.93	0.684
Women	Level 3	2772	62.1	1.72	0.70	4.19	0.234	0.91	0.61	1.37	0.661
	Level 2	1308	29.3	1.50	0.60	3.72	0.385	1.17	0.78	1.77	0.453
	Level 1	6	0.1	—	—	—	—	0.45	0.06	3.31	0.430

**Table 5:** Comparison between previous studies (HR for mortality, adjusted for age or demographic variables only).

	Participants	Source of		Men			Women	
		income data	Percentile	Hazard ratio	95% CI	Percentile	Hazard ratio	95% CI
The present study	65+	Government data	0–23.5*	1.00		0–3.3*	1.00	
(Japan)			78.7–99.6	1.53	1.28–1.82	70.1–99.6	1.54	0.97–2.45
								
Osler	20+	Government data	0–25*	1.00		0–25*	1.00	
(Denmark)			75–100	1.92	1.69–2.17	75–100	1.64	1.47–1.85
								
Manor**	45–69	Government data	0–25*	1.00				
(Israel)			75–100	1.61	1.45–1.79			
								
Bassuk***	65+	Interview survey	0–14.7*	1.00		0–5.9*	1.00	
(USA)		(response rate 82%)	74.6–100	1.72	1.20–2.48	49.0–100	2.13	1.34–3.38
								
Martikainen	30+	Government data	0–10*	1.00		0–10*	1.00	
(Finland)			80–90	2.02	1.96–2.08	70–80	1.47	1.42–1.53
						80–90	1.51	1.46–1.57

*Reference.

**Men only.

***Population of Connecticut (relatively similar in reference group percentile).
